# Relationship between Hg accumulation and diet in bats from Atlantic forest reserves

**DOI:** 10.1007/s10646-026-03067-y

**Published:** 2026-03-18

**Authors:** Cristiane dos Santos Vergilio, Diego Borges de Aguiar, Lucas Damásio, Diego Lacerda, Pedro Vianna Gatts, Eduardo de Sá Mendonça, Marcelo Gomes de Almeida, Beatriz Ferreira Araújo, Aureo Banhos, Carlos Eduardo de Rezende

**Affiliations:** 1 Departamento de Biologia, Centro de Ciências Exatas, Naturais e da Saúde, Universidade Federal do Espírito Santo, Alto Universitário, s/n, Alegre, Guararema, Alegre, 29500-000 Espírito Santo, ES Brazil; 2 Laboratório de Ciências Ambientais, Centro de Biociências e Biotecnologia, Universidade Estadual do Norte Fluminense Darcy Ribeiro, Campos dos Goytacazes, Avenida Alberto Lamego, 2000, Parque Califórnia, Campos dos Goytacazes, Rio de Janeiro, 28013-602 RJ Brazil; 3 Departamento de Agronomia, Centro de Ciências Agrárias e Engenharias, Universidade Federal do Espírito Santo, Alto Universitário, s/n, Alegre, Guararema, Alegre, 29500-000 Espírito Santo, ES Brazil; 4 Laboratoire Géosciences Environnement, Université Toulouse III – Paul Sabatier, 118 Route de Narbonne, Toulouse, 31400 France

**Keywords:** Brazil, Chiroptera, fur, mercury, stable isotopes, trophic guilds

## Abstract

Bats are valuable bioindicators of Hg contamination due to their wide representation across food webs, broad distribution, and high species richness worldwide. This study evaluated the influence of feeding habits and stable isotopes (δ¹³C and δ¹⁵N) on Hg concentrations in bats from a protected forest complex in southeastern Brazil. We collected road-killed bats (*n* = 100) along a highway crossing protected areas between 2012 and 2017. The mean Hg concentration (5.6 mg/kg; range: <0.003–71.3 mg/kg) found in the present study was higher than values reported for bats from reference sites, comparable to levels observed in the Peruvian Amazon (an area also affected by artisanal small-scale gold mining), and considerably lower than those measured in populations from highly contaminated regions. Hg concentrations did not vary with forearm length or sex but differed among feeding guilds, following the order: carnivorous > insectivorous > omnivorous > nectarivorous > frugivorous. Higher δ¹⁵N values and Hg concentrations observed in insectivorous bats likely reflect their diet of invertebrates, while lower δ¹⁵N values in frugivorous and nectarivorous bats are consistent with primarily plant-based diets. Differences in δ¹³C values among feeding guilds indicate variation in carbon sources and habitat use. In this study, 23% of bats exceeded the sub-clinical threshold of 5 mg/kg, a level associated with potential neurological and neurochemical effects. These findings demonstrate the influence of feeding guilds on δ¹³C, δ¹⁵N, and Hg concentrations in a tropical bat assemblage from the Atlantic Forest, highlighting their potential as sentinels for monitoring Hg contamination. Given their ecological diversity and varied feeding strategies, bats provide valuable insights into Hg sources and pathways within ecosystems. The susceptibility of insectivorous bats underscores the need to prioritize this group in future monitoring programs and conservation strategies, particularly in tropical ecosystems where high bat diversity coincides with intense anthropogenic pressures.

**Supplementary Information:**

The online version contains supplementary material available at 10.1007/s10646-026-03067-y.

## Introduction

Mercury (Hg) occurs naturally in the Earth’s crust and is continuously released into the environment through natural processes, such as volcanic eruptions and geothermal activity (Pirrone et al. 2010). However, anthropogenic sources—including fossil fuel combustion, waste incineration, and gold mining—greatly exceed natural emissions, resulting in elevated atmospheric Hg levels and its widespread global distribution (Pacyna et al. 2010). In the Hg cycle, elemental mercury (Hg⁰) is liquid at room temperature and becomes increasingly volatile with rising temperature. In the atmosphere, Hg⁰ vapor has a residence time of several months to years, which enables long-range transport (Cesário et al. 2017). Atmospheric Hg⁰ may be deposited directly or oxidized to Hg¹⁺ or Hg²⁺, which can bind with elements such as chlorine, sulfur, or oxygen to form inorganic Hg compounds, or with methyl groups to produce the organic forms methylmercury (MeHg) and dimethylmercury (Clarkson 1993). These Hg compounds are transported into both aquatic and terrestrial ecosystems, where they can be incorporated into the biota. Hg is highly toxic to humans and wildlife, with MeHg posing the greatest concern due to its capacity to bioaccumulate and biomagnify within food webs, thereby increasing the risk of exposure in top predators (Little et al. 2015; Becker et al. 2018).

Bats can be exposed to Hg through inhalation or contact with surfaces such as leaves, but diet is considered the primary pathway of contamination (Korstian et al. 2018). The order Chiroptera encompasses bat species with diverse feeding guilds that occupy a broad range of positions in food webs, including frugivores, nectarivores, insectivores, carnivores, piscivores, and sanguivores (Hernout et al. 2016a; Campbell et al. 2017; Clare and Oelbaum 2024). Collectively, these species provide essential ecosystem services such as pollination, insect population control, and seed dispersal (Ramírez-Fráncel et al. 2022). This ecological diversity, combined with key traits as (1) relatively long lifespans compared with other mammals of similar size (Wilkinson and South 2002), (2) wide distribution and high species richness worldwide (Yates et al. 2014), and (3) frequent coexistence with humans across urban, agricultural, and industrial landscapes (Zukal et al. 2015), makes bats a particularly suitable group for monitoring environmental contaminants. Hg exposure has been associated with a range of adverse effects in bats, including increased micronucleus frequency (Calao-Ramos et al. 2021), alterations in immune function—reported both as elevated (Becker et al. 2017) and reduced neutrophil counts (Becker et al. 2021)—as well as disruptions in neurochemical responses (Nam et al. 2012). Such effects may compromise individual health by increasing susceptibility to disease or predation, raising important questions about the broader ecological and physiological consequences of Hg contamination for bat populations.

Biological factors such as species-specific diet, foraging habitat, age, sex, reproductive status, body size, and molting cycles influence Hg accumulation in bats, often interacting with environmental exposure pathways (Nam et al. 2012; Yates et al. 2014; Zukal et al. 2015). Among these, sex plays an important role in Hg accumulation in bat fur, with females frequently showing higher concentrations than males (Yates et al. 2014; Kumar et al. 2018; Portillo et al. 2023). This difference is linked to a combination of biological and ecological factors. During reproduction, physiological changes mobilize stored Hg from tissues into circulation, with part of this burden incorporated into fur as a detoxification pathway. Although maternal transfer occurs via the placenta and, more importantly, through lactation, these processes are not always sufficient to substantially reduce the female’s total Hg load. Additionally, the increased energetic demands of gestation and lactation often require females to increase food intake, which may lead to higher dietary Hg exposure (Becker et al. 2017; Yates et al. 2014). Body size is another factor that may influence Hg accumulation. Forearm length is commonly used as a proxy for body size, as it correlates with both overall mass and wingspan (Meng et al. 2016; Davy et al. 2022). However, the relationship between forearm length and Hg concentrations in fur remains controversial. While most studies report no significant association, a positive correlation was observed in insectivorous *Nyctalus noctule* from the Russian taiga (Darwin Nature Reserve) with limited anthropogenic activity (Ivanova et al. 2024). These contrasting findings highlight the need for further investigation to clarify the extent to which biological factors influence Hg accumulation in bats.

Trophic ecology provides insights into how species interact with their environment and with each other through energy and nutrient flow, helping to identify key species, ecological roles, and dependencies among organisms (Perkins et al. 2014). Stable isotope ratios of carbon and nitrogen (δ¹³C and δ¹⁵N) allows the quantification of the dietary origin of assimilated (not just ingested) energy and nutrients (Hobson et al. 2000), overcoming the limitations of conventional analyses of feces and stomach contents (Crawford et al. 2008), as these methods reflect the animal’s diet only over a short period of time (Herrera et al. 2001). Therefore, the data of stable isotope analysis provide valuable insights into animal feeding habits and ecological niches (Lam et al. 2013). Given the wide diversity of feeding strategies in bats, isotopic data can help estimate the relative nutritional contributions of different food groups (Herrera et al. 2001). Trophic position data, along with metal quantification, can provide insights into the degree of bioaccumulation and potential biomagnification processes of contaminants within the food web. In bats, fur is an effective tissue for Hg analysis, as it provides a less invasive alternative to blood or tissue biopsies. Metal analysis of fur has been successfully applied in field-based studies of wildlife, including bats, to track pollution levels (Hernout et al. 2016b ). Fur is also widely used in stable isotope analyses, making it a versatile sample type for ecological and ecotoxicological studies (Baerwald et al. 2014; Segers and Broders 2015).

Tropical regions host the highest bat diversity worldwide, with species occupying a broad range of ecological niches and trophic levels (Becker et al. 2018). These areas also face multiple Hg sources, including coal combustion, gold mining, and deforestation (Costa et al. 2012). Rapid landscape transformation driven by agriculture, mining, dam construction, and urban expansion further intensifies Hg emissions and mobilization. Despite this elevated risk of exposure, most research on Hg in bats has focused on temperate regions, leaving major gaps in understanding bioaccumulation and biomagnification processes in tropical ecosystems. Expanding studies in these areas is therefore crucial to evaluate ecological risks, identify species- and habitat-specific vulnerabilities, and support both biodiversity conservation and public health strategies.

Brazil harbors approximately 13% of the world’s bat diversity, with 184 of the 1,500 recognized Chiroptera species occurring within its territory (Simmons and Cirranello 2025; Rodrigues et al. 2024). The highest species richness is found in the Amazon and Atlantic Forest biomes (Rodrigues et al. 2024). However, bat conservation in Brazil faces major threats, including plant exploitation, agricultural expansion, urbanization, energy production, mining, pollution, and climate change (Frick et al. 2020). Despite this remarkable diversity, there are still no records of Hg concentrations in bat assemblages across Brazilian biomes. Studies conducted in the Peruvian Amazon, in areas impacted by artisanal and small-scale gold mining, reported Hg levels in bats exceeding the threshold established for small mammals (10 mg/kg, Portillo et al. 2023)—conditions that are also present in parts of Brazil.

With this perspective, the present study investigated the influence of feeding habits on Hg concentrations in bats from a protected area complex in southeastern Brazil, a region surrounded by agriculture and intersected by a highway, which harbors the highest recorded bat diversity within the Atlantic Forest biodiversity hotspot (Damásio et al. 2021). The specific objectives were: (1) to quantify Hg concentrations across bat feeding guilds, with the prediction that Hg levels would differ among guilds, due to trophic position and diet composition; (2) to assess the influence of biological factors, specifically sex and body size (using forearm length as a proxy), on Hg accumulation, with the prediction that larger individuals and females (due to physiological and reproductive differences) would exhibit higher Hg concentrations; and (3) to infer which feeding guilds are most susceptible to Hg contamination by integrating stable isotope ratios of carbon and nitrogen (δ¹³C and δ¹⁵N), with the prediction that bats with higher δ¹⁵N values (indicating higher trophic positions) would show greater Hg accumulation, reflecting bioaccumulation and potential biomagnification within the food web.

## Materials and methods

### Study area

**Fig. 1:**
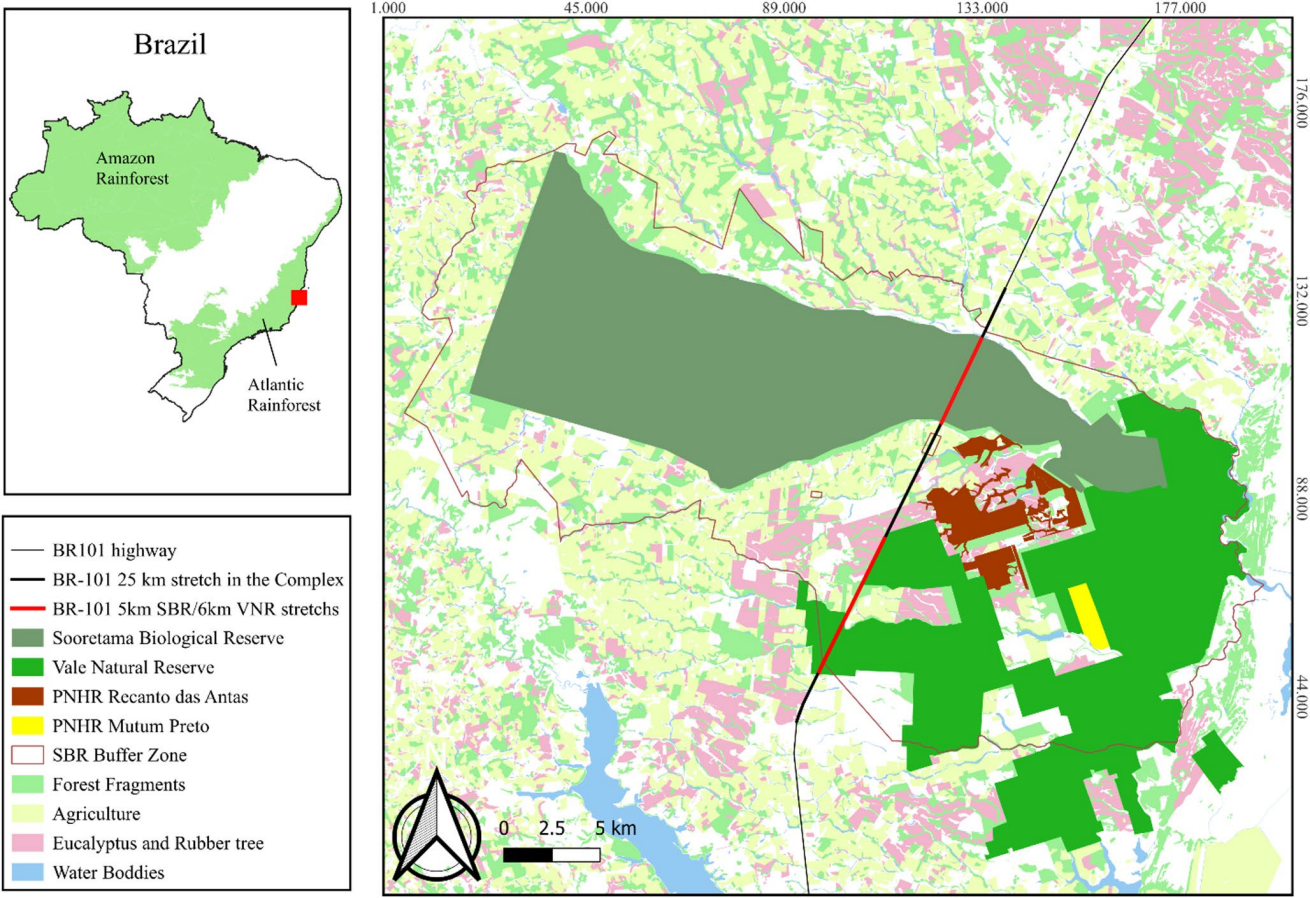
Map of the forest complex comprising the Sooretama Biological Reserve (SBR), Vale Nature Reserve (VNR), and the Private Natural Heritage Reserves (PNHR) Mutum Preto and Recanto das Antas. Bat sampling was conducted along a 25-km stretch of the highway crossing these reserves (highlighted black and red lines). The red lines show the road segments crossing the SBR (5.1 km) and the VNR (6 km). Map prepared by Lucas Damásio using ArcGIS software (ArcMap 10.8, version 10.7.0.10450; https://desktop.arcgis.com/en/)

The forest complex where bat samples were collected comprises the Sooretama Biological Reserve (SBR), a full protection unit covering 27,858 ha, its surrounding buffer zone, the Vale Nature Reserve (VNR; 23,500 ha), and two Private Natural Heritage Reserves (PNHRs): Mutum Preto (378.73 ha) and Recanto das Antas (2,201.60 ha). The SBR is a federally protected area, whereas the surrounding reserves are privately owned. Together, these areas encompass approximately 53,000 ha in northern Espírito Santo state, southeastern Brazil, spanning the municipalities of Sooretama, Vila Valério, Jaguaré, and Linhares (Fig. 1). This complex is part of the Atlantic Forest biome, with the SBR representing the largest remaining fragment of this vegetation type under formal protection in Espírito Santo (Rolim et al. 2016). This region is part of the Hileia Baiana, a subdivision of the Tabuleiro Forest and one of the most threatened phytophysiognomies within the Atlantic Forest (Faria et al. 2021; ICMBIO, 2020). It is also included in the Discovery Coast Atlantic Forest Reserves, which has been designated as a UNESCO World Heritage Site (UNESCO 2025).

Located within the Doce and Barra Seca River basins (28–65 m elevation), the reserves encompass a variety of ecosystems, including tall Tabuleiro Forest, muçununga (white-sand vegetation), native grasslands, swamps, and shallow valleys (Rolim et al. 2016). The region experiences a tropical climate, with annual rainfall ranging from 1,300 to 1,600 mm—approximately 80% of which occurs between October and March—and a mean annual temperature of 24.3 °C (Engel and Martins 2005; Saiter et al. 2017).

The forest complex is intersected by a 25-km stretch of the federal BR-101 highway, one of Brazil’s main north–south corridors along the eastern coast, carrying heavy traffic of both passenger and freight vehicles (Fig. 1). This highway section comprises a single lane in each direction, with a total width of approximately 15 m. The ecological impact of BR-101 has been documented across multiple taxonomic groups (Klippel et al. 2015; Srbek-Araujo et al. 2015; Banhos et al. 2021), with bats from various feeding guilds among the most affected (Damásio et al. 2021). The intense flux of vehicles of the highway also acts as source of Hg and other metal contamination due to road emissions from abrasion of paved surfaces, vehicles exhaust, engine fluids, and attrition from brakes and tires (Shi et al. 2010; Rodrigues et al. 2022). Additional environmental pressures in the region include surrounding monoculture plantations (e.g., eucalyptus, coffee, pepper, and papaya), which are intensively treated with pesticides (Rolim et al. 2016 ). The use of pesticides and both mineral and organic fertilizers also contribute to Hg release from agricultural activities (Sánchez-Báscones et al. 2016; Rashid et al. 2023).

### Bat sampling

The study was authorized under an environmental license from the Ministério do Meio Ambiente / Instituto Chico Mendes de Conservação da Biodiversidade (SISBIO protocol number 31762-5). Bat roadkill samples (*n* = 100) were collected through systematic monitoring along a 25-km stretch of the BR-101 highway intersecting the forest complex between the SBR and VNR. We conducted sampling between December 2012 and July 2017, during morning hours from 07:00 to 12:00. Two monitoring approaches were employed to collect data: on foot and by car. During months when both monitoring types overlapped, the methods were conducted alternately—on days when on-foot monitoring was performed, car-based monitoring was not conducted, and vice versa. Each monitoring session covered the entire highway section, beginning at one lane and ending at the other.

Bat roadkill were recorded in a spreadsheet, including a unique record number, kilometer mark, date, and time of observation. The coordinates of each collection site were obtained using a GPS device (Garmin eTrex 10), and carcasses were photographed. Carcasses of specimens found in suitable condition were collected for identification using morphological characters. Collected bats were placed in plastic bags, cataloged, and identified to the lowest possible taxonomic level using the identification keys by Díaz et al. (2016) and Gregorin and Taddei (2002). Species were assigned to feeding guilds following Díaz et al. (2016) and Damásio et al. (2021). Sex was determined, forearm length was measured as a biometric parameter, and fur samples were collected. Samples were stored at − 20 °C until analysis for Hg concentration and δ¹³C and δ¹⁵N stable isotopes. All animal handling procedures were approved by the Ethics Committee on the Use of Animals (CEUA), Alegre Campus – UFES, under protocol number 009/2019.

### Determination of Hg concentrations in bat fur

To evaluate Hg concentrations in bat guilds, fur samples were first washed in a 1:30 Extran^®^ Merck solution with ultrapure water, rinsed three times with ultrapure water, and air-dried at room temperature, following a procedure adapted from Korstian et al. (2018). For acid digestion, replicate fur samples were weighed and placed in tubes, followed by the addition of hydrogen peroxide (H₂O₂) and a 1:1 mixture of sulfuric and nitric acids (H₂SO₄:HNO₃). Samples were digested at 60 °C for solubilization, and 5 mL of 5% potassium permanganate (KMnO₄) was subsequently added. The resulting solution was titrated with 12% hydroxylamine hydrochloride (NH₂OH·HCl), filtered, and adjusted to a final volume of 15 mL with ultrapure water. Hg concentrations were then measured using a Hg Quick Trace M-750 CETAC-VARIAN analyzer, with a method detection limit of 0.003 mg/kg.

### Analysis of carbon and nitrogen stable isotopes

To assess which feeding habits are more susceptible to Hg contamination, stable isotope ratios of carbon and nitrogen (δ¹³C and δ¹⁵N) were measured in bat guilds. For the analysis, approximately 0.4–0.5 mg of fur was weighed into tin (Sn) capsules. Isotopic measurements were conducted using an organic elemental analyzer (Flash 2000) coupled to an isotope ratio mass spectrometer (Thermo Scientific Delta V Advantage). Method validation was performed using the standard OAS/isotope-low organic soil (elemental microanalysis), yielding a recovery of 94% and sampling precision above 95%. Detection limits were 0.05% for organic carbon (OC) and 0.02% for total nitrogen (TN). Analytical precision was assessed through triplicate analyses, resulting in a reproducibility of approximately ± 0.1‰ for δ¹³C and ± 0.2‰ for δ¹⁵N. All isotopic ratios are reported relative to international standards, with δ¹³C referenced to Pee Dee Belemnite (PDB) and δ¹⁵N referenced to atmospheric N₂.

### Statistics

Pearson’s correlation was used to assess the relationship between Hg concentrations and forearm length (morphological parameter). Differences in Hg levels between sexes and among feeding guilds for each isotope were analyzed using the nonparametric Kruskal-Wallis test, followed by Dunn’s post hoc test, as data did not meet assumptions of normality and homoscedasticity based on Shapiro-Wilk and Levene tests, respectively. Results were considered significant at *p* < 0.05. For each feeding guild, isotopic niche widths were quantified using standard ellipse areas (SEA) calculated with the SIBER package (Stable Isotope Bayesian Ellipses in R) (Jackson et al. 2011). For sample sizes below 30, the corrected standard ellipse area (SEAc) was used. All statistical analyses were performed in R software version 3.6.1 (“Action of the Toes”) for Windows via RStudio (Version 1.2.1335) (R Core Team, 2026 ).

## Results

In total, 39 bat species were identified, comprising one carnivorous, 18 insectivorous, two omnivorous, two nectarivorous, and 16 frugivorous species (Table 1). The mean Hg concentration across all samples was 5.6 mg/kg, ranging from < 0.003 to 71.3 mg/kg (Table 1), with the highest values occurring predominantly in insectivorous and carnivorous species, and lowest in nectarivorous and frugivorous. Species with the highest Hg concentrations included *Micronycteris microtis* (insectivorous), *Mimon crenulatum* (insectivorous), *Trachops cirrhosus* (carnivorous), *Phyllostomus sp.* (omnivorous), *Rhynchonycteris naso* (insectivorous), and *Molossus molossus* (insectivorous) (Table 1).

**Table 1:** Mean, minimum, and maximum total Hg concentrations (mg/kg), and number of individuals sampled for each bat species within their respective feeding guilds

Feeding Guild	Number	Hg (mg/kg)
Carnivorous	1	14.10
*Trachops cirhosus*	1	14.10
**Insectivorous**	**58**	**8.41 (0.62–71.3)**
*Centronycteris maximiliane*	2	6.95 (6.45–7.44)
*Lampronycteris brachyotis*	1	5.16
*Lophostoma brasiliense*	2	0.63 (0.62–0.64)
*Micronycteris megalotis*	1	3.34
*Micronycteris microtis*	2	26.93 (2.06–51.80)
*Micronycteris minuta*	1	2.27
*Micronycteris schmidtorum*	1	5.28
*Micronycteris sp.*	1	2.89
*Mimon crenulatum*	1	13,98
*Molossus molossus*	32	10.22 (1.82–71.34)
*Molossus rufus*	2	4.42 (3.41–5.42)
*Molossus sp.*	1	2.06
*Myotis sp.*	1	6.97
*Nyctinomops laticaudatus*	1	2.33 (0.73–3.93)
*Nyctinomops sp.*	1	3.49
*Rhynchonycteris naso*	1	11,25
*Saccopteryx bilineata*	5	3.26 (1.97–4.97)
*Saccopteryx leptura*	1	5.49
**Omnivorous**	**5**	**6.85 (0.84–16.70)**
*Phyllostomus hastatus*	3	2.33 (0.84–3.59)
*Phyllostomus sp.*	2	13.6 (10.5–16.70)
**Nectarivorous**	**8**	**1.19 (0.21–2.87)**
*Anoura geoffroyi*	2	0.72 (0.44–1.00)
*Anoura sp.*	1	0.21
*Driadonycteris capixaba*	1	1.46
*Glossophaga soricina*	4	1.60 (0.23–2.87)
**Frugivorous**	**28**	**0.36 (< 0.003–5.13)**
*Artibeus fimbriatus*	1	1.63
*Artibeus lituratus*	8	0.15 (0.04–0.27)
*Artibeus obscurus*	2	0.03 (0.02–0.03)
*Carollia perspicillata*	2	2.86 (0.60–5.13)
*Chiroderma sp.*	1	0.03
*Chiroderma villosum*	3	0.02 (0.01–0.02)
*Dermanura cinerea*	1	0.07
*Dermanura gnoma*	3	0.17 (0.03–0.46)
*Dermanura sp.*	1	0.08
*Platyrrhinus sp.*	1	0.02
*Platyrrhinus lineatus*	1	0.04
*Pygoderma bilabiatum*	1	0.06
*Sturnira lilium*	1	< 0.003
*Vampyressa pusilla*	2	0.30 (0.27–0.34)
**Total**	**100**	**5.56 (< 0.003–71.3)**

**Fig. 2:**
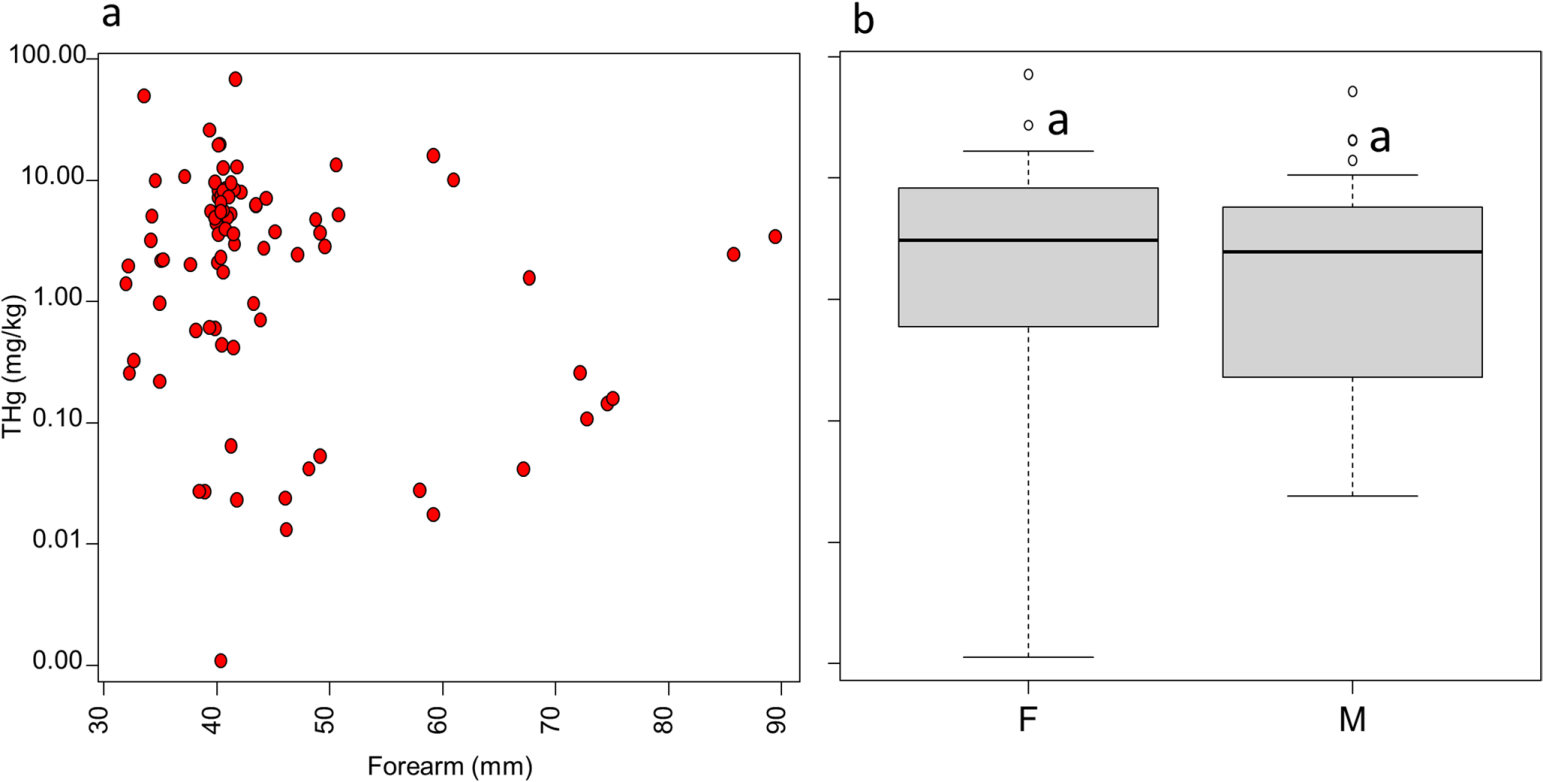
(**a**) Relationship between total Hg concentration and forearm length. (**b**) Comparison of Hg concentrations between sexes (F = female; M = male). The letter “a” indicates no statistically significant difference among groups, according to Dunn’s post hoc test (*p* < 0.05)

**Fig. 3:**
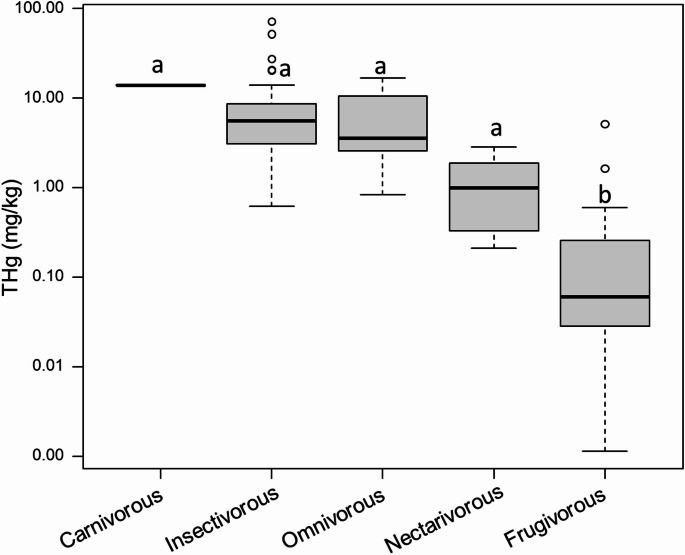
Differential Hg accumulation among bat feeding guilds. Different letters indicate statistically significant differences according to Dunn’s post hoc test (*p* < 0.05)

Hg concentrations showed no significant relationship with forearm length (Fig. 2a) or sex (Fig. 2b). However, significant differences were observed among feeding guilds, following the order: carnivorous (*n* = 1; 14.1 mg/kg) > insectivorous (mean = 8.41 mg/kg; range: 0.62–71.3 mg/kg) > omnivorous (mean = 6.85 mg/kg; range: 0.84–16.7 mg/kg) > nectarivorous (mean = 1.58 mg/kg; range: 0.23–2.87 mg/kg) > frugivorous (mean = 0.38 mg/kg; range: <0.003–5.13 mg/kg) (Fig. 3).

**Fig. 4:**
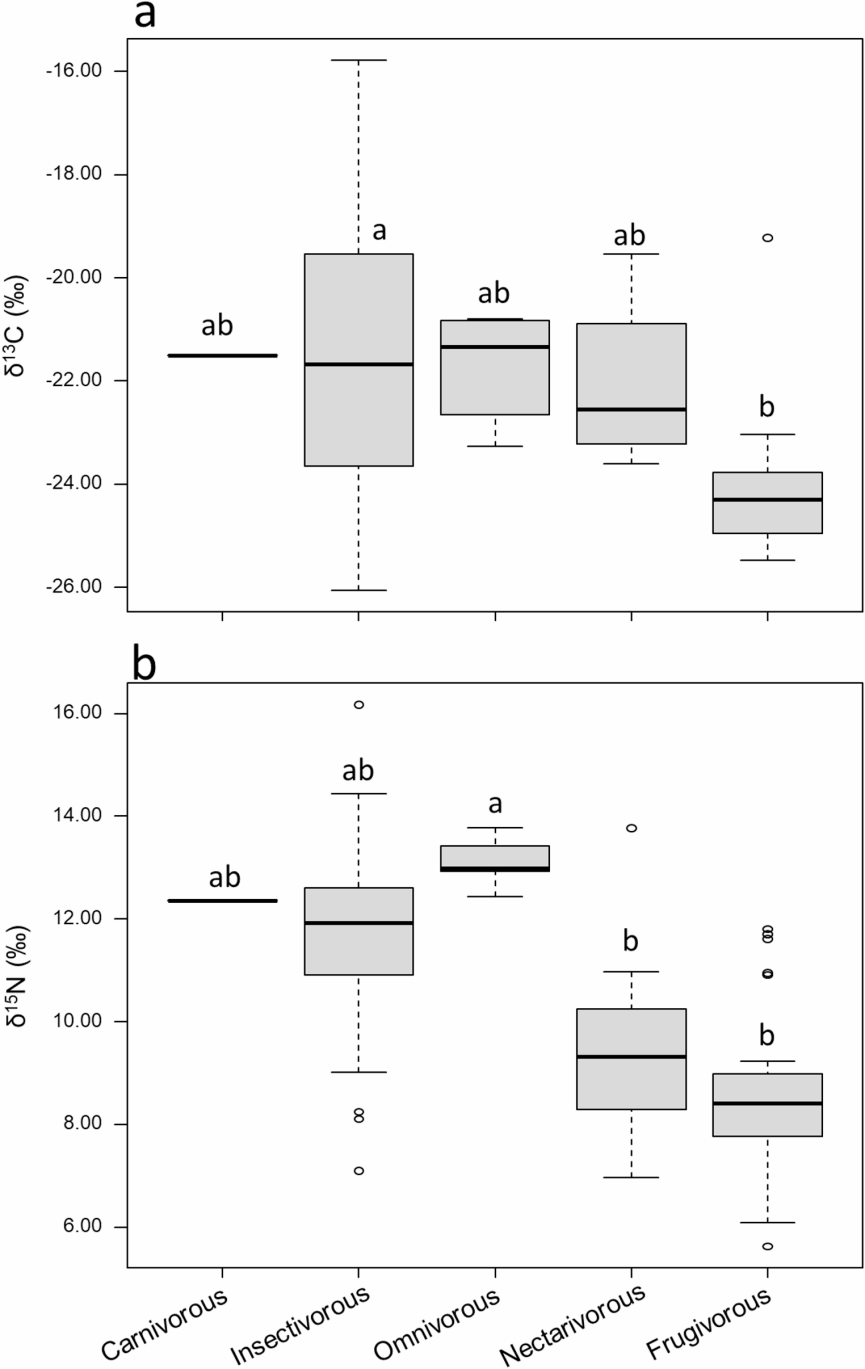
Variation in δ¹³C (a) and δ¹⁵N (b) values among bats from different feeding guilds. Different letters indicate statistically significant differences according to Dunn’s post hoc test (p < 0.05)

The feeding guild significantly influenced both δ¹³C and δ¹⁵N values (Fig. 4). δ¹³C values followed the order: frugivorous (–23.9‰) < nectarivorous (–23.0‰) < omnivorous (–21.8‰) < carnivorous (–21.5‰) < insectivorous (–20.0‰) (Fig. 4a). In contrast, δ¹⁵N values showed a different pattern, with frugivorous (8.6‰) < nectarivorous (10.0‰) < insectivorous (11.7‰) < carnivorous (12.4‰) < omnivorous (13.0‰) (Fig. 4b).

**Fig. 5:**
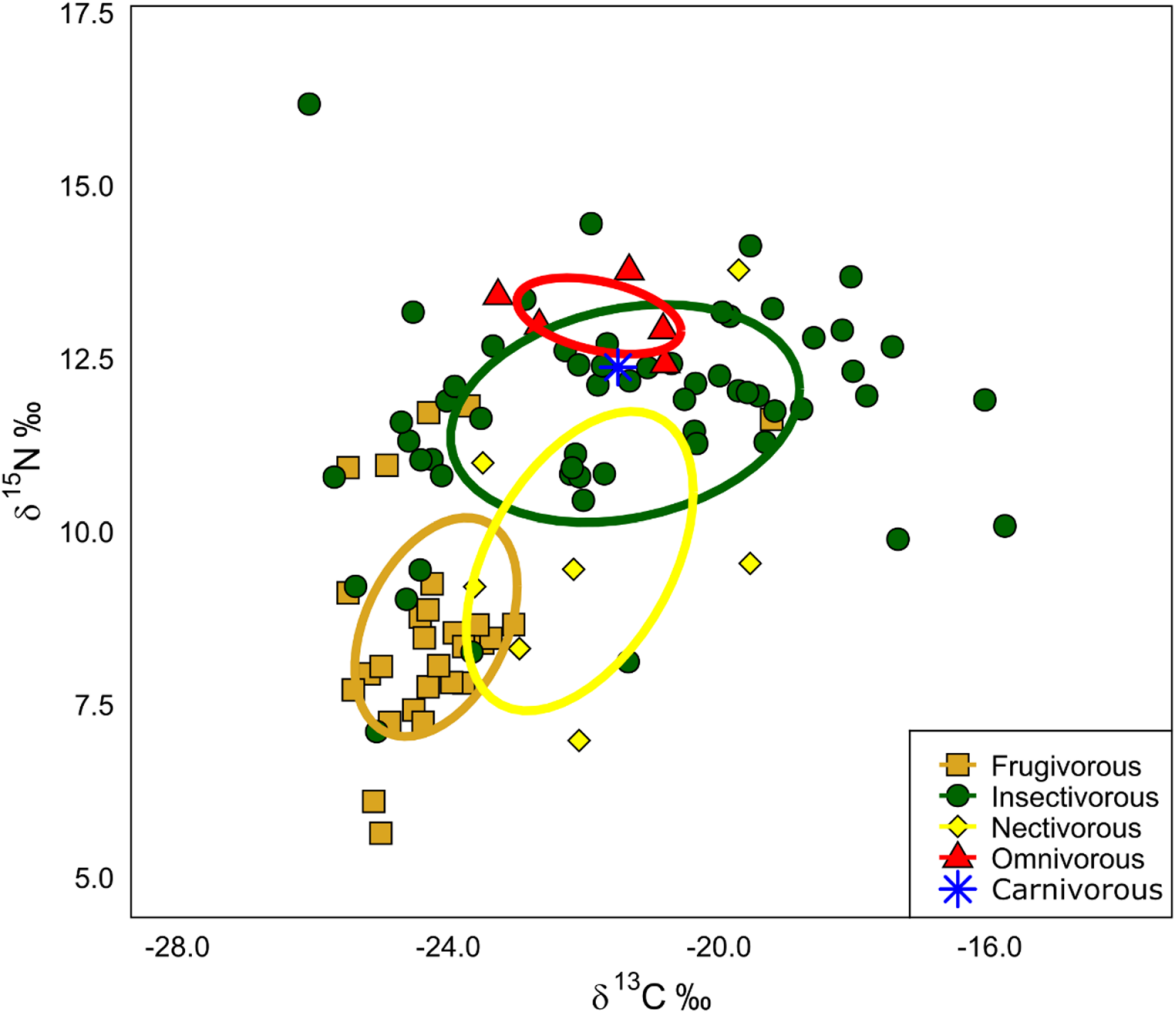
Biplot of δ¹³C and δ¹⁵N values showing the isotopic niche widths of different bat feeding guilds. Ellipses represent the standard ellipse areas (95% confidence intervals) for each guild

The δ¹³C–δ¹⁵N relationship also differed among feeding guilds, as shown by the analysis of isotopic niche width (Fig. 5). The standard ellipse areas revealed distinct positions and widths for each guild. Insectivores exhibited the broadest isotopic niche (SEA.B = 12.1‰), followed by nectarivores (SEA.B = 9.8‰), frugivores (SEA.B = 5.4‰), and omnivores (SEA.B = 1.8‰). The isotopic niche of insectivores overlapped with those of nectarivores and omnivores, whereas frugivores showed a more restricted niche, overlapping only with nectarivores (Table 2).

**Table 2:** Percentage overlap of isotopic niches among bat feeding guilds

Feeding guilds	Frugivorous	Insectivorous	Nectarivorous	Omnivorous
Frugivorous	-	0	33	0
Insectivorous	0	-	24	7
Nectarivorous	15	27	-	82
Omnivorous	0	42	2	-

## Discussion

The present study provides an overview of Hg concentrations in a bat assemblage from southeastern Brazil, contributing to understanding of Hg exposure in tropical bat populations. The study area harbors the highest recorded bat species diversity in the Atlantic Forest, with 71 species. It also exhibits the greatest abundance and diversity of road-killed bats worldwide, encompassing 47 species—predominantly insectivorous—that deliver essential ecological services, including the regulation of agricultural pests (Damásio et al. 2021). These findings are particularly relevant because, despite Brazil’s remarkable bat species richness, information on metal accumulation in this group remains scarce (Souza et al. 2020). To our knowledge, this study represents the first report of Hg concentrations in bat assemblages from Brazil. These data are essential for reinforcing conservation efforts in the Atlantic Forest biome, which is currently subjected to intense anthropogenic pressures such as deforestation, coal burning, agricultural expansion, pesticide use, gold mining, urban development, energy production, pollution, and climate change (Frick et al. 2020).

Bats sampled in this study were road-killed, influenced by several factors such as the attraction of insects to artificial lights, the open space created by the highway used by some species for foraging, and vehicle noise, which can disrupt flight and interfere with echolocation (Bhardwaj et al. 2017; Russo and Ancillotto 2015; Secco et al. 2017; Damásio et al. 2021). Additionally, the high traffic volume on the highway contributes to environmental contamination, including Hg and other metals, through road surface abrasion, vehicle exhaust, engine fluids, and wear from brakes and tires (Shi et al. 2010; Rodrigues et al. 2022). The forest complex where the bat samples were collected is surrounded by agricultural activities, which can serve as additional sources of environmental Hg contamination. Hg may be introduced through pesticide residues (Rashid et al. 2023) as well as through mineral and organic fertilizers (Sánchez-Báscones et al. 2016; Rashid et al. 2023). Furthermore, biomass burning—whether from crop residues or land clearing—releases Hg into the atmosphere (Béliveau et al. 2009; Huang et al. 2011). Soil management practices, including deforestation and irrigation, can also mobilize Hg already present in the soil (Almeida et al. 2005).

Fur Hg concentrations in the bat population examined in this study (mean = 5.6 mg/kg) were higher than those reported in reference sites (3.09 mg/kg - Moscow Barn, CA, USA - Nam et al. 2012; 0.08 mg/kg - Tambopata National Reserve in Peruvian Amazon, with no known mining activity - Portillo et al. 2023) and in areas influenced by agriculture in the Amazon plain of south-eastern Peru (1.33 ± 0.17 mg/kg – Carrasco-Rueda et al. 2020). They were similar to levels observed in the Peruvian Amazon, a region affected by artisanal small-scale gold mining (1.96–5.27 mg/kg – Portillo et al. 2023), yet considerably lower than values recorded in highly Hg-exposed bat populations from industrial sources in Virginia, USA (132 ± 94 mg/kg – Nam et al. 2012) and combined anthropogenic point sources in northeastern United States (52.46 mg/kg - Yates et al. 2014). Although differences in species composition and sample sizes exist between studies, these mean values provide useful context for assessing the potential risk of Hg contamination to bat populations globally. In the present study, 23% of the sampled bats exceeded the sub-clinical Hg threshold (> 5 mg/kg), levels that may induce neurological and neurochemical alterations, potentially impairing flight and foraging behavior (Nam et al. 2012; Zukal et al. 2015). Additionally, these individuals may experience compromised immune function, increasing susceptibility to bacterial infections (Becker et al. 2017), and may incur genotoxic damage due to Hg exposure (Calao-Ramos et al. 2021). Collectively, such effects could elevate the risk of mortality, including from roadkill.

Heavy metal concentrations can vary among species, sex, age, year of collection, and locality (Zukal et al. 2015). In the present study, however, fur Hg concentrations were not influenced by sex or forearm length. Sex-related differences in Hg accumulation may be linked to variations in food intake and body condition across life stages (juvenile, male in spermatogenesis, pregnant or lactating female), with reproductive females potentially more susceptible due to increased prey consumption during reproduction (Yates et al. 2014; Kumar et al. 2018). The limited sample size within each species in this study may have contributed to the lack of detectable correlation between sex and fur Hg levels. Regarding body size, previous studies have also reported no significant relationship between forearm length and Hg concentration in bats (Little et al. 2015), reinforcing that forearm length is not a reliable predictor of Hg accumulation in bat fur.

Differential Hg accumulation was observed among feeding guilds in the bats sampled in this study. The influence of feeding habits on Hg concentrations has been reported in other studies (Portillo et al. 2023; Carrasco-Rueda et al. 2020; Kumar et al. 2018). Higher Hg levels in insectivorous, omnivorous, and carnivorous species, coupled with lower levels in frugivorous bats, have also been documented in tropical ecosystems affected by artisanal and small-scale gold mining (Portillo et al. 2023; Carrasco-Rueda et al. 2020) as well as in other environments worldwide (Syaripuddin et al. 2014; Little et al. 2015; Becker et al. 2018; Moreno-Brush et al. 2017). Moreover, it is important to acknowledge potential sampling bias in the present study, as bats were collected primarily from roadkill. Consequently, some guilds may be underrepresented while others could be overrepresented due to factors such as foraging behavior, habitat use near roads, and differential susceptibility to vehicle collisions. These factors could affect the representativeness of each bat guild in the dataset.

Frugivorous and nectarivorous bats exhibit low Hg exposure due to their plant-based diets (Becker et al. 2018). In these species, Hg contamination primarily arises from atmospheric deposition, with secondary exposure occurring through contact with contaminated foliage (Zukal et al. 2015). In contrast, insectivorous bats are exposed predominantly through trophic bioaccumulation, whereby Hg is transferred from sediments, water, soil, and plants to insect larvae and adults, and ultimately to the bats (Hernout et al. 2013). Additionally, insectivorous bats that consume insects with aquatic larval stages or predators of aquatic insects facilitate the transfer of contaminants from aquatic sediments to terrestrial food webs (Becker et al. 2018).

The mean Hg concentrations in carnivorous (14.1 mg/kg), insectivorous (8.4 mg/kg), and omnivorous (6.8 mg/kg) bats exceed the sub-clinical threshold of 5 mg/kg, which may be sufficient to induce neurological and neurochemical effects (Nam et al. 2012), suggesting that these feeding guilds are particularly susceptible to Hg exposure. However, caution is warranted when comparing Hg levels across guilds due to differences in sample sizes; for example, the carnivorous guild in this study is represented by a single individual.

Feeding guilds also influenced the δ¹³C and δ¹⁵N signatures of the sampled bats, which was reflected in the distinct isotopic niche widths observed for each guild. Stable isotope analysis has been widely used to evaluate dietary sources and trophic relationships across different feeding guilds (Hobson 2019). In the present study, δ¹³C values ranged from -23.9‰ in frugivorous to -20.0‰ in insectivorous bats, while δ¹⁵N values ranged from 8.6‰ in frugivorous to 11.7‰ in insectivorous species. These values are consistent with previous studies on insectivorous bats (δ¹³C: -21.7 ± 0.1‰, δ¹⁵N: 8.8 ± 0.1‰ – Painter et al. 2009) and frugivorous bats (δ¹³C: -26.1 ± 0.3‰, δ¹⁵N: 4.4 ± 0.7‰ – Herrera et al. 2001; δ¹⁵N: 8.5 ± 0.1‰ – York and Billings 2009). This result is important for confirming the dietary patterns of each bat guild, as even when general feeding categories are established (e.g., frugivorous, insectivorous), isotopic signals can vary across regions, habitats, and seasons, reflecting local prey availability and ecosystem structure (Bearhop et al. 2004). Although feeding habits of most species are well known, stable isotope ratios offer a continuous and integrative measure of assimilated resources (Hobson et al. 2000), capturing variations within guilds that may affect contaminant uptake. In this study, isotopic values were consistent with expected dietary patterns, lower δ¹³C and higher δ¹⁵N values in insectivorous bats compared to frugivorous ones, confirming the trophic distinction among groups and supporting their use in interpreting Hg accumulation patterns.

It is important to note that, among bats, diet-related specializations do not preclude the continued exploitation of alternative food resources (Rex et al. 2010). In this context, the insectivorous bats included in our study comprise species with distinct foraging strategies (e.g., Molossidae and Phyllostomidae), a factor that can directly influence their isotopic signatures. Unlike many molossid bats that are more strictly aerial insectivores (Gregorin and Zanatta 2021), the phyllostomid insectivorous often forage in structurally complex habitats and exploit a broader range of food resources (Stevens 2022). The family Phyllostomidae is the most taxonomically diverse among bats in terms of number of genera and encompasses the greatest diversity of feeding strategies within the order Chiroptera (Berkovitz and Shellis 2018). Some phyllostomids species are strict diet specialists, but a considerable number species present mixed diets and will use both plant and animal food items, with geographical/temporal variation in resource availability (Monteiro and Nogueira 2011). Fruit consumption occurs to varying degrees across this family (Rojas et al. 2012), such that frugivorous bat species may complement their diets with animal protein, while insectivorous species may also opportunistically include fruits in their diet (Rex et al. 2010). This dietary flexibility directly influences carbon and nitrogen stable isotope signatures. Insects are rich in protein and low in digestible carbohydrates; therefore, even small contributions of insects to the diet can result in elevated nitrogen isotope (δ¹⁵N) values. In contrast, fruits and nectar are high in carbohydrates but low in protein and may be less strongly reflected in δ¹⁵N values (Rex et al. 2010). Consequently, isotopic signatures reflect assimilated resources rather than rigid feeding classifications. As a result, the dietary flexibility of some bat assembles can lead to overlapping isotopic signals among feeding guilds, reinforcing the importance of interpreting stable isotope data within the broader context of ecological interactions and nutritional composition.

Insectivorous bats exhibited the broadest isotopic niche, reflecting a more diverse and variable diet, whereas omnivorous and frugivorous bats showed narrower isotopic niches. However, the representation of the omnivorous guild in the sample was limited. The isotopic niche of insectivorous bats overlapped with those of nectarivorous and omnivorous species, while frugivorous bats displayed the most restricted niche, overlapping only with nectarivorous bats. Frugivorous bats generally exhibit lower Hg concentrations than insectivorous or piscivorous species, consistent with their diet and lower trophic position (Portillo et al. 2023; Carrasco-Rueda et al. 2020). In contrast, the elevated δ¹⁵N values and higher Hg concentrations observed in insectivorous bats reflect their diets’ greater integration of higher trophic levels, primarily due to the consumption of adult aquatic invertebrates that can retain elevated Hg levels accumulated during their larval and nymph stages in freshwater ecosystems (Kraus et al. 2014; Becker et al. 2018). These findings can guide the identification of the most sensitive species for monitoring programs and underscore the heightened susceptibility of insectivorous bats to metal contamination.

## Conclusion

This study provides the first assessment of Hg concentrations in bat assemblages from Brazil, contributing novel information from one of the most biodiverse regions for bats worldwide. By integrating Hg quantification with stable isotope analyses (δ¹³C and δ¹⁵N), we demonstrated that feeding guilds play a central role in Hg accumulation patterns, with carnivorous (limited sample size) and insectivorous bats exhibiting the highest concentrations, while nectarivorous and frugivorous species showed the lowest levels. These differences reflect trophic position and dietary pathways, reinforcing the importance of trophic ecology in understanding contaminant dynamics in wildlife.

Biological factors such as sex and body size did not significantly influence Hg concentrations, whereas dietary habits and trophic position were key determinants of exposure. The elevated Hg levels observed in a subset of individuals, including values exceeding subclinical thresholds, indicate potential risks for neurological and physiological effects, particularly among insectivorous bats. Given their wide dietary range, higher trophic integration, and sensitivity to environmental contamination, insectivorous bats emerge as especially valuable sentinels for Hg biomonitoring in tropical ecosystems.

These findings are particularly relevant given that, despite the high species richness of bats in the Atlantic Forest, the biome is currently under intense anthropogenic pressure. These data contribute for bat conservation in Brazil and highlight the urgent need for continued ecotoxicological monitoring in this group.

## Supplementary Information

Below is the link to the electronic supplementary material.

Supplementary Material 1

## Data Availability

Data is provided within the manuscript or supplementary information files.
